# UHPLC-MS/MS determination of varietal thiol precursors in Sauvignon Blanc grapes

**DOI:** 10.1038/s41598-017-13273-8

**Published:** 2017-10-13

**Authors:** Andreja Vanzo, Lucija Janeš, Franc Požgan, Špela Velikonja Bolta, Paolo Sivilotti, Klemen Lisjak

**Affiliations:** 1 Agricultural Institute of Slovenia, Department of Fruit Growing, Viticulture and Oenology and Central Laboratory, Hacquetova ulica 17, 1000 Ljubljana, Slovenia; 2 Faculty of Chemistry and Chemical Technology, University of Ljubljana, Večna pot 113, SI-1000 Ljubljana, Slovenia; 3 EN-FIST Centre of Excellence, Trg Osvobodilne fronte 13, SI-1000 Ljubljana, Slovenia; 4 University of Nova Gorica, Wine Research Centre, Glavni Trg 8, SI-5271 Vipava, Slovenia; 5 University of Udine, Department of Agricultural, Food, Environmental and Animal Sciences, via delle Scienze 206, 33100 Udine, Italy

## Abstract

Varietal thiol precursors in grapes are subject to metabolic changes during post-harvest treatments. Metabolic activity should therefore be limited after sampling to understand their biosynthesis in the berry and genetic regulation. In this study, berries were frozen in liquid nitrogen immediately after harvesting, transported in dry ice, stored briefly at −80 °C, cryo-milled and extracted without being thawed in cold methanol in a ratio of 1:4 (w/v). A UHPLC-MS/MS method for quantitative determination of the thiol precursors 3-*S*-glutathionylhexan-1-ol (G3MH), 3-*S*-cysteinylhexan-1-ol (Cys3MH), 4-*S*-glutathionyl-4-methylpentan-2-one (G4MMP) and 4-*S*-cysteinyl-4-methylpentan-2-one (Cys4MMP), glutathione, oxidized glutathione and L-methionine in grapes was developed. Reference material was provided through synthesis of precursors and their deuterium labelled analogues. The average thiol precursor content in grapes in 2013–15 was in the range 8–16 μg kg^−1^ for G3MH, 1–6 μg kg^−1^ for Cys3MH, 1–4 μg kg^−1^ for Cys4MMP and 0.3 μg kg^−1^ for G4MMP. In 2013 and 2014, the highest precursor content in mature Sauvignon Blanc grapes from vineyards located in Italy regarded G3MH, followed by Cys3MH, Cys4MMP and G4MMP. In 2015, G3MH was again the most abundant precursor, but followed by Cys4MMP, Cys3MH and G4MMP.

## Introduction

Sulfur-containing compounds which contribute to desirable fruity (grapefruit/citrus, passion fruit/tropical) aromas in Sauvignon Blanc are referred to as volatile or varietal thiols^1,2^. Among them, 3-mercaptohexan-1-ol (3MH, IUPAC: 3-sulfanylhexan-1-ol), 3-mercaptohexyl acetate (3MHA, IUPAC: 3-sulfanylhexyl acetate) and 4-mercapto-4-methylpentan-2-one (4MMP, IUPAC: 4-methyl-4-sulfanylpentan-2-one) have particularly low detection thresholds. Their presence is considered essential for creating unique varietal flavour attributes which contribute positively to wine. Varietal thiols are reportedly cleaved from their non-volatile precursors during fermentation with yeast β-lyases, which break the carbon-sulfur bond^3,4^. The most well-known thiol precursors in grapes are *S*-glutathione and *S*-cysteine conjugates: 3-*S*-glutathionylhexan-1-ol (G3MH), 3-*S*-cysteinylhexan-1-ol (Cys3MH), 4-*S*-glutathionyl-4-methylpentan-2-one (G4MMP) and 4-*S*-cysteinyl-4-methylpentan-2-one (Cys4MMP)^5–7^. Biosynthesis of thiol precursors is connected to glutathione (GSH) metabolism. GSH is an essential metabolite in plants, with multiple functions. A characteristic feature of GSH is its high concentration (it accumulates at millimolar concentrations) and its high level of reduction; in the absence of stress, for example, plant leaves maintain GSH:GSSG ratios of on average 20:1^8^. The biosynthesis of varietal thiols in grape berry is related to the conjugation of GHS and α,β-unsaturated carbonyl compounds by *S*-glutathione transferase as a part of the plant’s endogenous metabolism^7,9^. It has been suggested that the *S*-glutathione conjugate is then broken down by removing glutamic acid and glycine, resulting in the *S*-cysteine conjugate^7^.

The influence of thiol precursors on wine aroma has been studied extensively over the last two decades^2,10,11^ but there are still many questions that remain unanswered. The concentration of volatile thiols is thought to be related to the concentration of their precursors. However, only a small proportion of these precursors release the aromatic thiol during fermentation^12–14^. Sauvignon Blanc is a cultivar that can be strongly influenced in the vineyard and cellar to produce a range of different wine styles. While Sauvignon Blanc grapes are less aromatic, their corresponding wines can exhibit strong flavour, due to varietal thiol formation. Recent studies regarding Sauvignon Blanc thiol precursors have been oriented towards determining possible new precursors^15–17^, their localisation in the skin and juice of the grape berry^18,19^, their conversion to volatile thiols through the use of different yeast strains^3,20–22^, their evolution during grape ripening^23–26^, their fluctuation due to the time of harvesting (diurnal changes)^27^ and modifications taking place during harvest and post-harvest treatments^25,26,28,29^. It was found that in the presence of oxygen, part of the G3MH is produced in grape juice during prefermentative operations^25^. Furthermore, after transportation of machine-harvested Sauvignon Blanc grapes, a substantial increase in Cys3MH was observed in grape juice, together with limited formation of G3MH^28^. This was probably due to enzymatic degradation of G3MH into Cys3MH in unprotected grapes. Much lower G3MH content was found in grapes when enzymatic activity was prevented by freezing grapes in liquid nitrogen, pulverising and adding methanol/chloroform, as compared to household hand blender sample preparation^28^. Metabolic changes after freezing and thawing were also revealed in terms of the content of the G3MH precursor in grapes^26^.

Sample preparation techniques which prevent oxidation in effect prevent the modification of precursors after sampling. Therefore, proper sampling and preanalytical steps are important for reliable determination of thiol precursors content in grapes. To preserve the metabolite levels present at the time of sampling, a quenching protocol such as freezing of samples in liquid nitrogen must be quickly executed^30^. However, freezing destroys the cells, while thawing triggers all kinds of biochemical conversions. This should be prevented by blocking the metabolism during thawing, e.g. by extracting cryo-milled material with a strong solvent^30,31^. After that it would be ideal to use a direct injection approach. However, for complex plant tissues and metabolites at concentrations below certain levels, sample concentration is often a prerequisite. When a sample is concentrated, the integrity and performance of liquid chromatography mass spectrometry systems must be ensured. In the case of grapes, solid phase extraction offers an efficient method for the removal of interfering substances such as tannins and sugars.

The identification and quantification of thiol precursors in grapes is important in order to understand their biosynthesis in the berry and genetic regulation. The aim of this study was to design an experimental protocol for reliable determination of thiol precursors, reduced glutathione (GSH), oxidized glutathione (GSSG) and L-methionine (Met) in grape berries. Careful sampling in the vineyard, efficient extraction from grapes, cleaning and concentration of the extracts and robust UHPLC-MS/MS chromatographic conditions were set up. In order to provide pure reference materials, synthesis of the thiol precursors Cys4MMP, Cys3MH, G4MMP, G3MH and deuterium labelled analogues was undertaken. The content and distribution of the thiol precursors, Met, GSH, and GSSG were determined in Sauvignon Blanc grapes sampled in 5 different vineyards at harvest time in Friuli Venezia Giulia (Italy) in 2013–2015.

## Results and Discussion

### Synthesis

For the analysis of thiol precursors using UHPLC-MS/MS, analytical and deuterated internal standards of 4MMP and 3MH conjugates were synthesized by adopting published methods. The procedures in the literature were slightly modified as regards the ratios of reagents, reaction times and work-up of reaction mixtures. Since the final step in the preparation of cysteine conjugates (Cys3MH and Cys4MMP) was removal of the *tert*-butoxycarbonyl group using trifluoroacetic acid, the products were isolated as trifluoroacetate salts. Thiol precursors were characterised using ^1^H NMR and ^19^F NMR, spectroscopy and MS measurements (LC/TOF-MS and UHPLC-MS/MS).

### Grape sampling

In this study grapes were frozen with liquid nitrogen in the vineyard immediately after having been snipped (with scissors with pedicels), transferred to the laboratory in dry ice and stored at −80 °C before being analysed. On the contrary, in our preliminary study conducted in 2012 in the nearby vineyards of the Vipava Valley, Slovenia, grapes were not frozen immediately after they had been snipped. Instead, they were transported to a laboratory (2 hours by car at room temperature), stored at −80 °C and analysed following the same sample preparation procedure. In this case the average G3MH and Cys3MH contents were more than 10-fold higher than in the 2013 vintage with samples being immediately frozen and transported to the laboratory in dry ice (Supplementary material: Table S1). This is in accordance with the findings of others who reported the G3MH formation and enzymatic degradation of G3MH into Cys3MH during transportation in case enzymatic activity was not prevented immediately^28^.

### Grape extract preparation and purification

Cryogenic milling, rapid transfer of frozen pulverised grapes into 100% ice-cold, deoxygenated methanol in a ratio of 1:4 (w/v), short extraction at room temperature, centrifugation and direct injection was the protocol used. When the thiol precursors (usually this was only G4MMP) were below analytical limits for evaluation by direct injection, grape extracts were concentrated and purified by the use of solid phase extraction. Schematic outline of sample preparation steps is shown in Supplementary material: Fig. S1. The absence of the oxidation during sampling and sample preparation steps has been shown by the high redox status (GSH:GSSG ratio) in all analysed grape samples (for details please see chapter Analysis of grapes). Interestingly, higher G3MH contents were found when cryo-milled grapes were thawed before being transferred to organic solvent, in comparison to cryo-milled grapes immediately transferred (data not shown). These results point to the G3MH formation after berry damage if enzymatic activity was not prevented. This was already reported in some previous studies^26,28^.

### UHPLC-MS/MS analytical method

**Figure 1:**
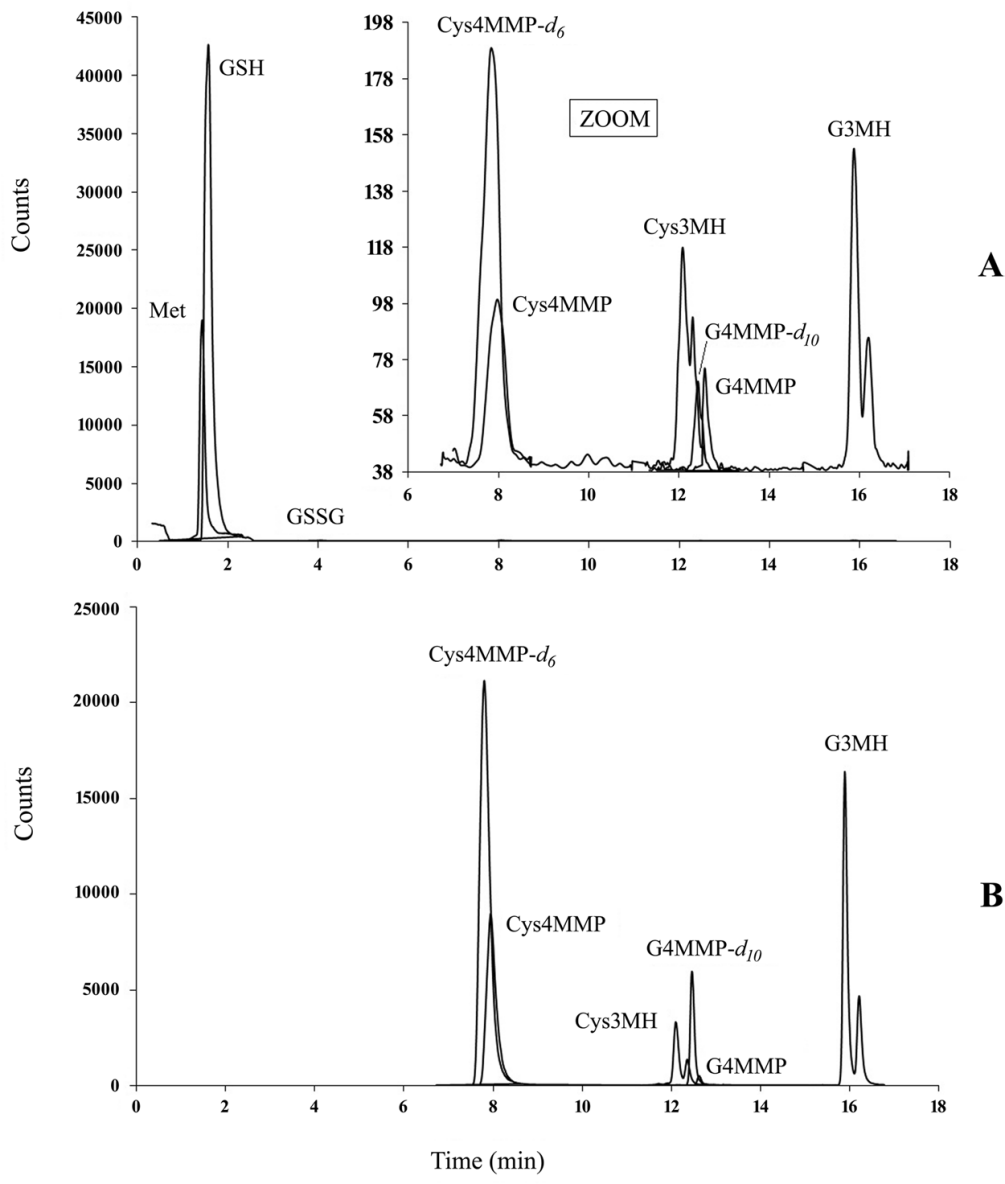
ESI + selected reaction monitoring (SRM) chromatogram of methionine, glutathione, oxidized glutathione, thiol precursors and deuterium labelled analogues of MMP conjugates in Sauvignon Blanc grape methanol extract – direct injection (**A)** and ESI + SRM chromatogram of thiol precursors and deuterium labelled analogues of MMP conjugates in purified and concentrated Sauvignon Blanc grape extract (**B**).

The UHPLC-MS/MS method enabled good separation, detection and quantification of Met, GSH, GSSG and thiol precursors, together with Cys4MMP and G4MMP deuterium analogues in grape extracts (Fig. 1A). This method also allows quantification of cysteine (amino acid, a GSH  precursor), but the data were omitted, since they were below the detection limits in the grape extracts (cryo-milled grape diluted in methanol 1:4, w/v). To determine thiol precursors (usually G4MMP) below the analytical limits (LOQ) in the grape extract, grape extracts were purified and concentrated according to the procedure described. Thiol precursors were then quantified considering recoveries of deuterated internal standards. Recovery of G4MMP-*d*_10_ was used for quantification of G4MMP, whereas recovery of Cys4MMP-*d*_6_ was used for Cys4MMP. 3MH precursors were usually above the LOQ with direct injection of grape extracts. If this was not the case, recovery of Cys4MMP-*d*_6_ was used to determine Cys3MH, whereas G4MMP-*d*_10_ was used for G3MH. After the purification process, the precursors were concentrated, whereas GSH, GSSG and Met were not present in the concentrate (Fig. 1B). Diastereoisomers of Cys3MH and G3MH were integrated as one peak (Fig. 1A,B).

The analytical method would also make it possible to measure the content of thiol precursors in grape juice and must. Must was diluted in methanol in the ratio 1:4 (to limit enzymatic activity after sampling), centrifuged, filtered and directly injected. The higher content of 3MH precursors found in must in comparison to grapes allowed easy determination of both 3MH precursors, through direct injection of must five times diluted in methanol. A purification procedure was used to determine 4MMP precursors, however with only a hydrophobic cartridge for sugar removal. Must diluted in methanol was evaporated under reduced pressure at 38 °C, the residue was diluted in 5 mM H_2_SO_4_ (4 mL) and quantitatively loaded onto a Strata column following the purification procedure.

### UHPLC-MS/MS method validation

The linearity of the detector response for thiol precursors, Met, GSH and GSSG was verified in synthetic grape must (sugar content 200 g L^−1^, pH 3.2, titratable acidity 7 g L^−1^ as tartaric acid, 125 mg L^−1^ of mixture: (+)-catechin, (−)-epicatechin, procyanidin B1 and procyanidin B2) diluted in methanol (1:4 v/v). Nine concentration levels (between 0.1 and 350 μg/L for thiol precursors) were injected in six repetitions for each level. Linearity was determined using the F-test and multiple linear regressions. The limit of detection and limit of quantification were calculated by considering signal to noise S/N = 3 and S/N = 10 respectively (Table 1).

**Table 1:** Linearity, limit of detection (LOD), limit of quantification (LOQ) for thiol precursors, GSH, GSSG and Met.

Compound	Range (µg L^−1^)	R^2^	LOD (µg L^−1^)	LOQ (µg L^−1^)
Cys4MMP-*d*_6_	0.10–348.50	0.9994	0.10	0.29
Cys4MMP	0.10–297.40	0.9955	0.10	0.30
Cys3MH	0.09–297.36	0.9978	0.09	0.28
G4MMP-*d*_10_	0.13–381.00	0.9928	0.04	0.13
G4MMP	0.12–348.90	0.9934	0.01	0.03
G3MH	0.11–330.00	0.9959	0.06	0.17
**Compound**	**Range (mg L** ^**−1**^ **)**	**R** ^**2**^	**LOD (mg L** ^**−1**^ **)**	**LOQ (mg L** ^**−1**^ **)**
Met	0.01–10.00	0.9932	0.01	0.03
	10.00–100.00	0.9874		
GSH	1.01–25.28	0.9916	0.06	0.20
	25.28–101.1	0.9832		
GSSG	1.01–25.2	0.9945	0.10	0.32
	25.2–100.8	0.9830		

The repeatability and reproducibility of the analytical method with the use of purification/concentration procedure were determined within a 5-day period; two grape extracts were prepared each day from cryo-milled grapes stored at −80 °C. Grape extracts were purified and concentrated according to the procedure. Standard deviation of repeatability and standard deviation of reproducibility were calculated. Dispersion of results was checked with Cochran’s test and outliers with Grubbs’ test. The uncertainty of repeatability and reproducibility were calculated by multiplying the standard deviation of repeatability and the standard deviation of reproducibility by Student’s t-factor, with 4 degrees of freedom and a 95% confidence level (t95; 4 = 2.776). The analytical method had shown a satisfactory repeatability and reproducibility (Table 2).

**Table 2:** Standard deviations of repeatability and reproducibility for thiol precursors with the use of purification/concentration procedure.

	Cys4MMP-*d*_6_ μg L^−1^	Cys4MMP μg L^−1^	Cys3MH μg L^−1^	G4MMP-*d*_10_ μg L^−1^	G4MMP μg L^−1^	G3MH μg L^−1^
Means of levels	2.33	2.00	2.19	3.38	2.18	6.55
Standard deviation of repeatability for the level (*s*_*r*_)	0.11	0.10	0.15	0.15	0.17	0.22
Standard deviation of reproducibility for the level (*s*_*R*_)	0.24	0.18	0.21	0.24	0.24	0.40
Repeatability limit (*r*)	0.43	0.38	0.58	0.60	0.66	0.87
Reproducibility limit (*R*)	0.95	0.69	0.82	0.93	0.93	1.56
Uncertainty of repeatability (*U*_*r*_)	0.31	0.27	0.41	0.42	0.45	0.62
Uncertainty of reproducibility (*U*_*R*_)	0.67	0.49	0.58	0.66	0.66	1.10

### Analysis of grapes

In 2013–15, Sauvignon Blanc grape samples were collected at harvest time in five representative vineyards in Friuli Venezia Giulia (Italy) and the Met, GSH, GSSG and thiol precursor content was determined (Table 3).

**Table 3:** L-methionine (Met), glutathione (GSH), oxidized glutathione (GSSG) and thiol precursor content in Sauvignon Blanc grapes from Friuli Venezia Giulia (Italy) in 2013, 2014 and 2015.

**Location**	Met	GSH	GSSG	GSH: GSSG	Cys4MMP	Cys3MH	G4MMP	G3MH	Total prec.
	mg kg^−1^	mg kg^−1^	mg kg^−1^	ratio	μg kg^−1^	μg kg^−1^	μg kg^−1^	μg kg^−1^	μg kg^−1^
**2013**									
L1	2.07	111.54	1.63	68.49	4.78	15.10	0.19	20.86	40.92
L2	3.93	114.31	2.77	41.27	3.88	5.21	0.21	17.33	26.63
L3	2.56	111.46	1.64	67.96	4.68	2.30	0.42	12.16	19.55
L4	5.16	109.71	1.95	56.26	4.87	2.80	0.42	12.68	20.77
L5	2.89	105.22	1.99	52.87	2.26	5.44	0.17	18.05	25.91
Average	**3.32**	**110.45**	**2.00**	**57.37**	**4.09**	**6.17**	**0.28**	**16.21**	**26.76**
CV* (%)	37.12	3.04	23.34	19.81	26.81	83.99	45.57	22.90	31.77
**2014**									
L1	2.08	26.39	1.55	17.03	0.34	1.61	0.06	10.16	12.16
L2	3.43	66.68	2.22	30.04	0.69	0.67	0.11	12.08	13.55
L3	3.86	83.85	0.69	121.52	2.93	3.12	0.86	10.42	17.33
L4	3.22	33.61	2.01	16.72	1.33	2.00	0.15	2.95	6.43
L5	2.98	46.84	0.48	97.58	0.87	0.53	0.20	4.47	6.08
Average	**3.11**	**51.48**	**1.39**	**56.58**	**1.23**	**1.58**	**0.28**	**8.02**	**11.11**
CV (%)	21.32	46.07	56.06	87.29	82.36	66.74	119.03	50.33	43.39
**2015**									
L1	2.40	55.97	0.70	79.96	4.48	0.18	0.16	11.03	15.85
L2	2.12	55.24	0.42	131.52	1.43	0.75	0.18	3.64	6.00
L3	4.75	94.88	0.81	117.14	3.76	4.28	0.46	14.18	22.68
L4	2.75	65.79	1.14	57.71	2.87	0.85	0.15	12.54	16.41
L5	4.41	95.37	1.95	48.91	4.85	0.90	0.71	19.84	26.31
Average	**3.29**	**73.45**	**1.00**	**87.05**	**3.48**	**1.39**	**0.33**	**12.25**	**17.45**
CV (%)	36.79	27.53	58.59	41.61	39.44	117.89	74.97	47.80	44.44

*Coefficient of variation.

The amino acid Met (source of organic S and N available to yeasts during fermentation) content in grapes was generally similar in different seasons, with the lowest variability of data in 2014. As regards GSH content, in 2013 significantly higher values were measured, being on average 110 mg kg^−1^. Interestingly, the mean G3MH content was also highest in 2013, and this could be linked to the higher GSH content, which could participate in G3MH biogenesis. The GSSG values were not significantly different for the seasons, and high GSH:GSSG ratios in grapes showed that no oxidative stress occurred in grape berries at the time of sampling and during sample preparation steps.

On average, G3MH was the most abundant and G4MMP the least abundant thiol precursor in Sauvignon Blanc grapes from all locations in all the years investigated. Cys3MH was the second most abundant precursor in 2013 and 2014, whereas in 2015 the average Cys4MMP content was higher than Cys3MH (Table 3). The average thiol precursor content in grapes in the three years was in the range 8–16 μg kg^−1^ for G3MH, 1–6 μg kg^−1^ for Cys3MH, 1–4 μg kg^−1^ for Cys4MMP and 0.3 μg kg^−1^ for G4MMP. Many researches have shown concentrations of thiol precursors in grape juice or must. However, to our knowledge there are only few data with the quantification of glutathionyilated and cysteinylated precursors of 3MH and 4MMP in grapes. In the study of Capone *et al*.^26^ 3MH precursor diastereoisomers were determined during ripening of five Sauvignon blanc clones located in Australia (Adelaide Hills). Grapes were protected with 20 mg kg^−1^ SO_2_, homogenised with a household hand blender, centrifuged and the supernatant was used for analyses. At the time of harvest approximately 200 μg kg^−1^ of G3MH and 30  μg kg^−1^ of Cys3MH were found in different Sauvignon Blanc clones. According to their findings, G3MH was more abundant than Cys3MH in grapes; however, they reported more than 10 fold higher G3MH and Cys3MH content than that found in this study. It was later proved that there was an increase in both G3MH and Cys3MH concentrations during the transportation of grapes without antioxidants (SO_2_ and ascorbic acid)^28^. Besides, there was a dramatic decrease in G3MH concentration if enzymes were inhibited by freezing grapes in liquid nitrogen, grinding and extraction in organic solvent^28^. This suggested that G3MH could be formed during transport and processing as a result of berry damage and acknowledged the role of enzymatic conjugation^32^. Roland *et al*.^18^ reported on average c. 20–110 μg kg^−1^ of G3MH, 15–80 μg kg^−1^ of Cys3MH and less than 10 μg kg^−1^ of G4MMP in grape pericarp (skin and flesh) samples from the Touraine, Sancerre and Montpellier regions respectively. They found differences in both thiol precursor content and the distribution as regards Sauvignon Blanc origin^18^. In their study grapes were cut with scalpel to remove pedicels and seeds. The skin was separated from the pulp then frozen using liquid nitrogen, crushed in liquid nitrogen, powdered, stored at −80 °C and extracted in solution containing sodium metabisulfite and benzenesulfinic acid before analysis^18^.

In 2013–2015 the ratio between G3MH and Cys3MH in grapes was 2.63, 5.08 and 8.81 respectively, whereas the ratio between G4MMP and Cys4MMP was 0.07, 0.23 and 0.09 respectively. There are reports that the ratio between glutathionylated and cysteinylated MH precursors could be considered as specific for each variety^12^. However, changing ratios in the grape berry in different seasons were observed in this study.

Statistical analysis of the data (Table 4) revealed no differences between locations (all vineyards were in Friuli Venezia Giulia within a distance of 1–15 km from the meteorological station; Supplementary Material: Fig. S2). On the other hand, the particular meteorological conditions of the three seasons investigated led to several differences in both basic maturation parameters and the accumulation of varietal thiol precursors (Table 4). As regards the seasons, 2013 was only characterised by low temperatures during May and June, with high rainfall in May and a lack of water in July (Supplementary Material: Fig. S3). In contrast, the following season in 2014 was characterised by low temperatures in July and August, while rainfall was abundant from July till harvest. The 2015 season had the highest temperatures from May to September, with rainfall well distributed throughout the season. The differences between seasons, briefly described in the previous paragraphs, could explain the modifications in grape quality characteristics. The Brix values were significantly higher in 2013 and 2015, while in contrast titratable acidity was very high and pH lower in the colder year in 2014. Although significant differences were ascertained only for Cys4MMP and total precursors, the total amount and the relative percentages of all precursors in grapes were not constant for different years. This is in accordance with other authors, who have reported that G3MH was most abundant in grapes, and that differences in concentration were related to the particular meteorological conditions at the location where Sauvignon Blanc was produced^18^.

**Table 4:** Effect of location and season on basic maturation parameters, methionine (Met), glutathione (GSH), oxidized glutathione (GSSG) and thiol precursor content in Sauvignon Blanc grapes from Friuli Venezia Giulia (Italy) in 2013–2015.

Factor	TSS	TA	pH	Met	GSH	GSSG	Cys 4MMP	Cys 3MH	G4MMP	G3MH	Total prec.
	(°Brix)	(g/L)		mg kg^−1^	mg kg^−1^	mg kg^−1^	μg kg^−1^	μg kg^−1^	μg kg^−1^	μg kg^−1^	μg kg^−1^
**Location**											
L1	18,80	9,45	3,10	2,18	64,63	1,29	3,20	5,63	0,14	14,01	22,98
L2	22,21	7,59	3,20	3,16	78,74	1,80	2,00	2,21	0,16	11,02	15,40
L4	20,50	8,08	3,19	4,59	96,15	1,15	3,85	3,40	0,58	12,42	20,26
L3	21,04	7,27	3,31	2,84	70,29	1,60	2,96	1,72	0,24	9,22	14,13
L5	18,98	8,62	3,17	3,43	82,48	1,47	2,66	2,29	0,36	14,12	19,43
*Sign. F*	*ns*	*ns*	*ns*	*ns*	*ns*	*ns*	*ns*	*ns*	*ns*	*ns*	*ns*
**Year**											
2013	21,91 a	7,64 b	3,26 a	3,32	110,45 a	2,00	4,09 a	6,17	0,28	16,21	26,76 a
2014	18,03 b	10,10 a	3,05 b	3,11	51,48 b	1,39	1,23 b	1,58	0,28	8,02	11,11 b
2015	20,99 ab	6,87 b	3,27 a	3,29	73,45 b	1,00	3,48 a	1,39	0,33	12,25	17,45 ab
*Sign. F*	*	*	*	*ns*	**	*ns*	*	*ns*	*ns*	*ns*	*

Data were analysed with mixed-model ANOVA (location, fixed factor; year, random factor - ns, not significant; *p < 0.05; **p < 0.01). Different letters represent significant differences in Tukey’s HSD test (p < 0.05).

## Conclusion

The analytical method presented in this study offers the accuracy and sensitivity required to understand viticultural and climatic factors impacting the biosynthesis of thiol precursors in grape berries. Thiol precursors are located in both grape juice and grape skin. Cryogenic milling of grapes and extraction in cold methanol aimed to preserve the metabolite levels present at the time of sampling. The possibility of direct evaluation of thiol precursors, methionine, glutathione and oxidized glutathione after just one extraction step offers a fast and more precise analytical approach, by avoiding complex sample preparation steps. As regards G4MMP, which was impossible to detect with direct injection, a purification/concentration procedure with deuterated internal standard was used.

Sauvignon Blanc grapes were sampled at the time of harvest at five different locations in north-eastern Italy in the period 2013–2015. It was found that the G3MH precursor always dominated in grapes, whereas G4MMP was always less abundant. Cys3MH was the second most abundant precursor, followed by Cys4MMP in 2013 and 2014, whereas in 2015 Cys4MMP was more abundant than Cys3MH. The average content and relative proportions of the individual precursors changed in different years.

## Methods

### Chemicals and reagents

L-Glutathione (reduced), glutathione oxidized, L-methionine, *trans*-2-hexenal, acetonitrile, sodium borohydride (NaBH_4_), ethanol, *N*-(*tert*-butoxycarbonyl)-L-cysteine (Boc-Cys-OH), triethylamine, sodium sulfate, triethylsilane, trifluoroacetic acid, 4-methylpent-3-en-2-one (mesityl oxide), mesityl oxide-d_10_, sodium carbonate, acetic acid, hydrochloric acid, (+)-catechin, (−)-epicatechin, (−)-epicatechin gallate, procyanidin B1 and procyanidn B2 were obtained from Sigma-Aldrich (St. Louis, MO, USA). Diethyl ether, pyridine, dichloromethane, 1,4-dioxane, pentane and ethyl acetate were obtained from Merck (Darmstadt, Germany). Formic acid (LC-MS, Fluka), methanol (LC-MS, Chromasolv, Sigma-Aldrich) and ultra-pure water of Milli Q gradient (EMD Millipore, Billerica, MA, USA) were used for chromatography. For grape grinding under cryogenic conditions (−196 °C) a M20 mill from IKA (Staufen, Germany) was used.

### Synthesis of thiol precursors

#### 3-*S*-glutathionylhexan-1-ol (G3MH)

G3MH was prepared in two step synthesis^33^. A mixture of acetonitrile (5.5 mL) and water (2.5 mL) was purged with argon, and L-glutathione (750 mg, 2.44 mmol), *trans*-2-hexenal (300 μL, 2.56 mmol) and pyridine (393 μL, 4.88 mmol) were then added. After stirring in an inert atmosphere at room temperature for 45 h, the mixture was concentrated under reduced pressure, water (10 mL) was added to the residue and aqueous solution was washed with dichloromethane (3 × 10 mL). The organic layers were discarded. Sodium borohydride (190 mg, 7.32 mmol) in water (7 mL) was added dropwise to the aqueous layer at 0 °C. After stirring at room temperature for 3 h, the mixture was acidified with 1 M HCl to pH 2–3, concentrated under reduced pressure and dried with a vacuum pump at room temperature. The solid residue was dissolved in water (3.5 mL) and purified on a C18 semi-prep column with mobile phases of (A) water and (B) acetonitrile; the gradient was: 1–5% B from 0–20 min, 5–15% B from 20–40 min and 15–40% B from 40–60 min. Fractions from 26–35 min were evaporated to provide pure G3MH as a white solid (55 mg, 6%).

^1^H NMR (500 MHz, D_2_O): δ = 0.86 (m, 3 H), 1.38 (m, 2 H), 1.54 (m, 2 H), 1.72 (m, 1 H), 1.84 (m, 1 H), 2.14 (m, 2 H), 2.45–2.58 (m, 2 H), 2.78–2.88 (m, 2 H), 3.05 (dd, *J* = 13.5, 5 Hz, 1 H), 3.66–3.75 (m, 2 H), 3.78 (t, *J* = 6.5 Hz, 1 H), 3.93 (s, 2 H), 4.54 (m, 1 H) ppm (Supplementary Material: Fig. S4).

High resolution mass spectra (HRMS) (UHPLC/TOF-MS), [M + H]^+^ calculated for C_16_H_29_N_3_O_7_S 408.1799 Da, detected 408.1800 Da (Fig. S5).

#### 3-*S*-cysteinylhexan-1-ol (Cys3MH)

Cys3MH was synthesized in a three-step reaction starting from *trans*-2-hexenal and Boc-protected L-cysteine^21,34^ with slightly modified protocols to reduce the formation of undesirable by-products and the presence of impurities in the final product^35^. *trans*-2-Hexenal was added in three portions (470 μL (4.05 mmol) at t = 0 h, 340 μL (2.93 mmol) at t = 12 h and 180 μL (1.55 mmol) at t = 24 h) to a solution of *N*-(*tert*-butoxycarbonyl)-L-cysteine (430 mg, 1.94 mmol) and triethylamine (620 μL, 4.45 mmol) in 1,4-dioxane (9 mL). After stirring in an inert atmosphere at room temperature for additional 24 h, the mixture was evaporated under reduced pressure and water (10 mL) was added to the residue. The aqueous solution was acidified with 5% HCl to approximately pH 3 and washed with dichloromethane (2 × 10 mL). The combined organic phases were washed with water (10 mL) and evaporated under reduced pressure. The oily residue was rinsed with pentane (10 × 5 mL), evaporated under reduced pressure and dissolved in phosphate buffer (Na_2_HPO_4_/NaH_2_PO_4_, 10 mL, pH 8, 1 M), to which sodium borohydride (250 mg, 6.61 mmol) in water (4 mL) was added dropwise. After stirring at room temperature for 3 h, the mixture was diluted with water (3 mL) and washed with dichloromethane (3 × 10 mL). The organic layers were discarded and the aqueous layer was acidified with 25% acetic acid to approximately pH 6 and extracted with ethyl acetate (3 × 20 mL). The combined organic phases were dried over anhydrous Na_2_SO_4_ and evaporated under reduced pressure. The residue was dissolved in dichloromethane (6 mL), and triethylsilane (135 μL, 0.845 mmol) and trifluoroacetic acid (856 μL, 11.19 mmol) were added successively at 0 °C. After stirring in an inert atmosphere at 0 °C for 20 min, the volatiles were evaporated under reduced pressure. The residue was dissolved in water (5 mL) and washed with ethyl acetate (3 × 20 mL). The organic layers were discarded and the aqueous layer was evaporated under reduced pressure. The oily residue was treated with diethyl ether (5 mL), evaporated again under reduced pressure and dried overnight with a vacuum pump at room temperature to give white product Cys3MH as trifluoroacetate salt (150 mg, 23%).

^1^H NMR (500 MHz, D_2_O): δ = 0.88 (t, *J* = 7.5 Hz, 3 H), 1.40 (m, 2 H), 1.57 (m, 2 H), 1.71 (m, 1 H), 1.86 (m, 1 H), 2.88 (m, 1 H), 3.00–3.18 (m, 2 H), 3.71 (m, 2 H), 4.06 (m, 1 H) ppm (Fig. S6).

^19^F NMR (470 MHz, D_2_O): δ = –75.6 ppm (Fig. S7).

HRMS (UHPLC/TOF-MS), [M + H]^+^ calculated for C_9_H_19_NO_3_S 222.1158 Da, detected 222.1149 Da (Fig. S8).

#### 4-*S*-glutathionyl-4-methylpentan-2-one (G4MMP)

G4MMP was prepared in a base-catalysed reaction between glutathione and mesityl oxide as described^6^ with some modifications. Water (4 mL) was purged with argon, and L-glutathione (150 mg, 0.488 mmol), pyridine (267 μL, 3.308 mmol) and mesityl oxide (84 μL, 0.734 mmol) were then added. After stirring in an inert atmosphere at room temperature for 48 h, the mixture was diluted with water (10 mL) and washed with dichloromethane (5 × 10 mL). The organic layers were discarded and the aqueous layer was evaporated under reduced pressure. The solid residue was suspended in a mixture of water/ethanol (1 mL/13 mL). After cooling for 24 h the precipitate was filtered off to give pure G4MMP as a white solid (55 mg, 28%).

^1^H NMR (300 MHz, D_2_O): δ = 1.46 (s, 6 H), 2.22 (m, 2 H), 2.30 (s, 3 H), 2.59 (m, 2 H), 2.90 (s, 2 H), 3.00 (dd, *J* = 13.0, 8.4 Hz, 1 H), 3.15 (dd, *J* = 13.0, 5.4 Hz, 1 H), 3.88 (t, *J* = 6.3 Hz, 1 H), 4.03 (s, 2 H), 4.64 (dd, *J* = 8.4, 5.4 Hz, 1 H) ppm (Fig. S9).

HRMS (UHPLC/TOF-MS), [M + H]^+^ calculated for C_16_H_27_N_3_O_7_S 406.1642 Da, detected 406.1637 Da (Fig. S10).

#### [^2^H_10_] 4-*S*-glutathionyl-4-methylpentan-2-one (G4MMP-*d*_10_)

For the preparation of deuterium labelled G4MMP-*d*_10_ mesityl oxide-*d*_10_ was used instead of mesityl oxide^13^. Water was purged with argon, then L-glutathione (150 mg, 0.488 mmol) and pyridine (267 μL, 3.308 mmol) were added. Mesityl oxide-*d*_10_ was added to the mixture in three portions: 50 μL (0.437 mmol) at t = 0 h, 20 μL (0.175 mmol) at t = 8 h and 15 μL (0.131 mmol) at t = 20 h. After stirring in an inert atmosphere at room temperature for an additional 24 h, the mixture was diluted with water (10 mL) and washed with dichloromethane (4 × 10 mL). The organic layers were discarded and the aqueous layer was evaporated under reduced pressure. The solid residue was suspended in a mixture of H_2_O/EtOH (1 mL/13 mL). After cooling for 24 h the precipitate was filtered off to give pure G4MMP-*d*_10_ as a white solid (52 mg, 26%).

^1^H NMR (500 MHz, D_2_O): δ = 2.14 (m, 2 H), 2.45–2.56 (m, 2 H), 2.81 (m, 1 H), 2.93 (dd, *J* = 13.0, 8.5 Hz, 1 H), 3.08 (dd, *J* = 13.0, 5.0 Hz, 1 H), 3.79 (t, *J* = 6.3 Hz, 1 H), 3.93 (s, 2 H), 4.56 (dd, *J* = 8.5, 5.0 Hz, 1 H) ppm (Fig. S11).

HRMS (UHPLC/TOF-MS), [M + H]^+^ calculated for C_16_H_17_N_3_O_7_SD_10_ 416.227 Da, detected 416,2289 Da (Fig. S12).

#### 4-*S*-cysteinyl-4-methylpentan-2-one (Cys4MMP)

Cys4MMP was prepared in two-step synthesis according to the literature^34^ with some modifications. Boc-Cys-OH (200 mg, 0.904 mmol) and mesityl oxide (385 μL, 3.366 mmol) were added to a solution of Na_2_CO_3_ (101 mg, 0.953 mmol) in water (6 mL). After stirring in an inert atmosphere at room temperature for 48 h, the mixture was washed with diethyl ether (4 × 10 mL). The organic layers were discarded and the aqueous layer was acidified with 25% acetic acid to approximately pH 5 and extracted with ethyl acetate (2 × 10 mL). The combined organic phases were dried over anhydrous Na_2_SO_4_ and evaporated under reduced pressure. The residue was dissolved in dichloromethane (2 mL), and triethylsilane (40 μL, 0.250 mmol) and trifluoroacetic acid (360 μL, 4.704 mmol) were added successively at 0 °C. After stirring in an inert atmosphere at 0 °C for 20 min, the volatiles were evaporated under reduced pressure. The residue was dissolved in water (5 mL) and washed with ethyl acetate (3 × 10 mL). The organic layers were discarded and the aqueous layer was evaporated under reduced pressure. The oily residue was treated with diethyl ether (10 mL), evaporated again under reduced pressure and dried overnight with a vacuum pump at room temperature to give white product Cys4MMP as trifluoroacetate salt (60 mg, 20%).

^1^H NMR (500 MHz, D_2_O): δ = 1.41 (s, 6 H), 2.24 (s, 3 H), 2.86 (m, 2 H), 3.09 (dd, *J* = 7.5, 14.0 Hz, 1 H), 3.21 (dd, *J* = 14.0, 4.2 Hz, 1 H), 4.11 (m, 1 H) ppm (Fig. S13).

^19^F NMR (470 MHz, D_2_O): δ = –75.6 ppm (Fig. S14).

HRMS (UHPLC/TOF-MS), [M + H]^+^ calculated for C_9_H_17_NO_3_S 220.1002 Da, detected 220.1015 Da (Fig. S15).

#### [^2^H_6_] 4-*S*-cysteinyl-4-methylpentan-2-one (Cys4MMP-*d*_6_)

The procedure was the same as described for the synthesis of Cys4MMP except that mesityl oxide-*d*_10_ was used instead of mesityl oxide^34^. Boc-Cys-OH (200 mg, 0.904 mmol) and mesityl oxide-*d*_10_ (385 μL, 3.366 mmol) were added to a solution of Na_2_CO_3_ (101 mg, 0.953 mmol) in water (6 mL). After stirring in an inert atmosphere at room temperature for 48 h, the mixture was washed with diethyl ether (4 × 10 mL). The organic layers were discarded and the aqueous layer was acidified with 25% acetic acid to approximately pH 5 and extracted with ethyl acetate (2 × 10 mL). The combined organic phases were dried over anhydrous Na_2_SO_4_ and evaporated under reduced pressure. The residue was dissolved in dichloromethane (2 mL), and triethylsilane (40 μL, 0.250 mmol) and trifluoroacetic acid (360 μL, 4.704 mmol) were added successively at 0 °C. After stirring in an inert atmosphere at 0 °C for 20 min, the volatiles were evaporated under reduced pressure. The residue was dissolved in water (5 mL) and washed with ethyl acetate (3 × 10 mL). The organic layers were discarded and the aqueous layer was evaporated under reduced pressure. The oily residue was treated with diethyl ether (10 mL), evaporated again under reduced pressure and dried overnight with a vacuum pump at room temperature to give white product Cys4MMP as trifluoroacetate salt (97 mg, 31%).

^1^H NMR (500 MHz, D_2_O): δ = 2.22 (s, 3 H), 2.84 (m, 2 H), 3.05 (dd, *J* = 13.5, 7.5 Hz, 1 H), 3.18 (dd, *J* = 13.5, 4 Hz, 1 H), 3.96 (m, 1 H) ppm (Fig. S16).

^19^F NMR (470 MHz, D_2_O): δ = –75.7 ppm (Fig. S17).

HRMS (UHPLC/TOF-MS), [M + H]^+^ calculated for C_9_H_11_NO_3_SD_6_ 226.1379 Da, detected 226.1402 Da (Fig. S18).

### NMR and UHPLC/TOF-MS analysis of synthesized standards

Nuclear magnetic resonance (NMR) spectra were recorded on a Bruker Avance III 500 spectrometer operating at 500 MHz for proton and 470 MHz for fluorine nuclei. Chemical shifts were recorded as δ values in *parts per million* (ppm). Spectra were acquired in deuterium oxide (D_2_O) at ambient temperature. ^1^H NMR spectra were referenced against the water signal at 4.79 ppm. HRMS were recorded on 6230 Agilent UHPLC/TOF-MS (Palo Alto, CA, USA) in positive mode using electrospray ionization (ESI, Jet-Stream). Stock standard solutions of individual compounds were prepared in methanol:water 1:1 (v/v) and stored at −20 °C for grape analysis.

### Grape sampling

Sampling was carried out on 3 September 2013, 28 August 2014 and 31 August 2015 in 5 vineyards located in Friuli Venezia Giulia (north-eastern Italy). The locations were selected within the three most important designations of controlled origin (DOC) regions where Sauvignon Blanc is produced, namely Friuli Isonzo, Friuli Colli Orientali & Ramandolo and Collio. The following locations were selected: Dolegna (L1), Rosazzo (L2), Romans d’Isonzo (L3), Capriva (L4) and Gorizia (L5). All the Sauvignon Blanc vineyards in Friuli Venezia Giulia were planted with clone R3 and on rootstock (SO4). Meteorological data from Capriva del Friuli (ARPA FVG - OSMER, http://www.meteo.fvg.it) were used for characterisation of the three seasons, being considered representative of the five locations (Fig. S2). The distance between the weather station and the vineyards ranges from 1 to 15 km.

Grapes were sampled in the morning on the same day at all locations. In each season three replicates of 50 berries were collected in three different plots of 25 randomly selected vines within the vineyard. Berries were snipped with scissors with the pedicels from different bunches and vine positions. They were collected randomly from the top, the centre and the tip of several clusters, both exposed to the sun and in shade. For the purpose of the analysis, a subsample of 50 berries was separated from three replicates. Grape samples were supposed to be collected at technological maturity in all years. While in 2013 and 2015 this rule was followed, in the cooler year of 2014, the low temperatures and excessive rain characterising the last months before harvest (Fig. S3) did not allow complete maturation of the grapes; thus the samples were collected on the last possible date, just before harvest.

In order to prevent metabolic changes, grapes were frozen with liquid nitrogen immediately after snipping. Samples were transferred to the laboratory in dry ice and stored at −80 °C before being analysed. Analysis of samples was performed each year within a month of sampling.

### Grape extraction

Methanol was chosen as an appropriate solvent for the extraction of thiol precursors from grapes, because it is known to be effective in terms of protein precipitation, enzyme inactivation and efficient extraction of polar to semi non-polar metabolites from tissues^31,36–38^. Cold, deoxygenated methanol was found to be effective in inhibiting oxidase enzymes and preventing glutathione depletion^39^. The authors reported that optimum recovery of glutathione content in grape juice and wine was obtained when either the sample of grape juice or wine was mixed in cold deoxygenated methanol in the ratio 1:9 (v:v)^39^. To limit enzymatic and chemical reactions, the solvent was deoxygenated by streaming nitrogen and cooled to −20 °C before being used. Since thiol precursors are located in the skin and pulp of the berry^18,19^, a homogeneous sample was provided by cryogenic grinding of grape berries with liquid nitrogen.

### Optimisation of extraction

To find the proper mass to solvent ratio, Sauvignon Blanc grapes were cryogenically ground and extracted in 100% methanol and 90% methanol in water with sample weight to solvent volume (w/v) ratios of 1:9 and 1:4 respectively. Four repetitions were recorded for each set. Extracts were centrifuged and filtered through a 0.22 μm PVDF filter from Millipore (Billerica, MA, USA). Met, GSH, GSSG and thiol precursors were analysed using UHPLC-MS/MS. Extraction of grapes in 100% cold, deoxygenated methanol in a ratio of 1:4 (w/v) was chosen as the best option for precursors (Supplementary Material, Table S2). Assuming that mature grapes contain approximately 80% water, the aqueous part after extraction in 100% methanol (in a ratio of 1:4) was *c*. 17%, which improved the solubility of hydrophilic compounds such as Met, GSH and GSSG.

### Extraction procedure

Pulverised frozen grapes (10 g) were rapidly transferred into 40 mL of ice-cold methanol, spiked with Cys4MMP-*d*_6_ and G4MMP-*d*_10_ (5 µL, 25 mg L^−1^ in 50% aqueous methanol), vortexed, extracted for 10 min at room temperature and centrifuged for 5 min at 4 °C, 10000 × g. The volume was adjusted with cold methanol. An aliquot of 200 μL was filtered through a 0.22 μm PVDF filter from Millipore (Billerica, MA, USA) and directly injected onto UHPLC-MS/MS. The remaining supernatant was purified and concentrated as described below. Met, GSH, GSSG and thiol precursors present above analytical quantification limits (ten times signal to noise) were quantified by direct injection.

### Concentration and purification of grape extracts

When the thiol precursors (usually only G4MMP) were below analytical limits for evaluation by direct injection, grape extracts were concentrated and purified to eliminate tannins and sugars which would impact the integrity of mass spectrometry. Extraction was performed using a Dowex 50WX4–100 ion exchange resin in a micro-column, with a modified method as previously described for grape juice and must^25^. Methanol was evaporated from the extracts under reduced pressure at 38 °C, the residue was diluted in water (4 mL) and quantitatively loaded onto 160 mg of ion exchange resin, previously conditioned with water (1.2 mL), followed by 2 M HCl (1.2 mL) and water (6 mL). The sample was washed with water (6 mL) and eluted with 4 mL of 1 M ammonium buffer (NH_4_H_2_PO_4_). The eluent was subsequently loaded onto a hydrophobic cartridge (6 mL, 500 mg, Strata SDB-L), previously conditioned with methanol (6 mL) and 5 mM H_2_SO_4_ (6 mL). The cartridge was washed with 5 mM H_2_SO_4_ (5 mL) and dried under a flow of nitrogen. Thiol precursors were eluted with methanol (5 mL). Methanol was evaporated to dryness under reduced pressure at 38 °C and the residue was immediately redissolved in 200 µL mixture of methanol:water 1:1, filtered through a 0.22 μm PVDF filter and analysed using UHPLC-MS/MS.

To assess recovery of thiol precursors during the purification process, grape extracts (n = 6) were spiked with a thiol precursor mixture (5 µL, 25 mg L^−1^ in 50% aqueous methanol), vortexed and centrifuged. A small aliquot was filtered and directly injected into UHPLC-MS/MS. Grape extracts were then purified according to the procedure (see above) and recovery of purification was determined with regard to direct injection. The average recoveries (±standard error of the mean, n = 6) of Cys4MMP-*d*_6_, Cys4MMP, Cys3MH, G4MMP-*d*_10_, G4MMP, and G3MH from grape extracts during purification were 69.0 ± 2.3%, 66.2 ± 3.0%, 76.8 ± 8.9%, 76.4 ± 8.1%, 71.4 ± 8.7% and 73.3 ± 6.9% respectively.

### UHPLC-MS/MS analysis

Analysis was carried out using a 1290 infinity UHPLC system coupled to a 6460 triple quadrupole mass spectrometer (Agilent Technologies). Samples were kept at 4 °C during analysis and the injection volume was 5 μL. Separation was performed on a 100 mm × 2.1.mm, 1.8 μm column (Acquity HSS T3, Waters) maintained at 40 °C. Flow was set to 0.4 mL/min, mobile phase A was 0.1% formic acid, and B 0.1% formic acid in methanol. The linear gradient started at 0% B, to 2% B in 5 min, to 35% B in 15 min, to 100% B in 1 min, isocratic at 100% B for 3 min, to 0% B in 1 min, and 3 min post time at 0% B. Analysis was performed in positive mode using electrospray ionization (ESI, Jet-Stream) and selected reaction monitoring (SRM) acquisition. Nitrogen was used as the carrier gas and the source parameters were: gas temperature 250 °C, gas flow 6 L min^−1^, nebulizer 241 KPa, sheath gas heater 375 °C, sheath gas flow 10 L min^−1^, capillary 4000 V. Three mass transitions were selected for each compound: one for the quantifier and two for qualifiers (Table 5).

**Table 5:** Chromatographic retention times and selected reaction monitoring conditions.

Compound	Retention time (min)	Precursor ion (m/z)	Quantifier ion (m/z)	Qualifier ions (m/z)	
Met	1.43	150.1	56.1	104.0	133.0
GSH	1.56	308.1	162.2	179.3	116.1
GSSG	3.68	613.2	355.0	484.0	231.0
Cys4MMP-*d*_6_	7.84	226.1	122.2	105.3	168.0
Cys4MMP	7.97	220.1	122.2	105.1	99.2
Cys3MH	12.33	222.1	83.2	205.1	101.1
G4MMP-*d*_10_	12.48	416.2	269.5	287.4	179.1
G4MMP	12.64	406.3	259.4	179.3	162.2
G3MH	16.05	408.2	162.2	279.4	262.4

## Electronic supplementary material

UHPLC-MS/MS determination of varietal thiol precursors in Sauvignon Blanc grapes
