# Supplementary material for: UHPLC-MS/MS determination of varietal thiol precursors in Sauvignon Blanc grapes
Source: Sci Rep. 2017 Oct 13;7:13122. doi: 10.1038/s41598-017-13273-8 (PMC5640626; doi:10.1038/s41598-017-13273-8)
Supplement: Supplementary file 1 — UHPLC-MS/MS determination of varietal thiol precursors in Sauvignon Blanc grapes [file 41598_2017_13273_MOESM1_ESM.pdf]

UHPLC-MS/MS determination of varietal thiol precursors in Sauvignon Blanc grapes

Andreja Vanzo[1], Lucija Janeš[1], Franc Požgan[2,3], Špela Velikonja Bolta[1], Paolo Sivilotti[4,5], Klemen Lisjak[1,*]

SUPPORTING INFORMATIONS

Results and discussion

Grape sampling

Location	Methionine	GSH	GSSG	G3MH	Cys3MH	G4MMP	Cys4MMP	Total precursors
	(mg/kg)	(mg/kg)	(mg/kg)	(μg/kg)	(μg/kg)	(μg/kg)	(μg/kg)	(μg/kg)
Vrtovin	1,2	55,5	2,2	54,9	3,7	0,1	1,5	60,1
Dobravlje	1,5	62,6	1,2	26,5	3,4	0,1	3,9	33,8
Planina	4,2	72,0	2,6	54,5	3,4	0,1	0,9	58,9
Lože	2,7	82,4	2,8	21,4	3,6	0,2	2,8	28,0
Vipava	28,1	98,1	2,3	36,5	3,5	0,3	2,7	43,0
**Average Vipava 2013**	**7,5**	**74,1**	**2,2**	**38,7**	**3,5**	**0,2**	**2,4**	**44,8**
CV	1,5	0,2	0,3	0,4	0,0	0,7	0,5	0,3
**Average Vipava 2012**	no data	no data	no data	**456,6**	**38,8**	**0,7**	**0,5**	**496,6**

Table S1. Contents of thiol precursors in Sauvignon Blanc grapes sampled in Vipava valley (Slovenia) in 2013 (samples were frozen in liquid nitrogen in the vineyard and transported to laboratory in dry ice) and the average content of thiol precursors in Sauvignon Blanc grapes sampled in Vipava valley (Slovenia) in 2012 (samples were not frozen immediately and were transported 2 h by car at room temperature).

Lisjak, K., Janeš, L., Velikonja Bolta, Š., Bavčar, D., Čuš, F., Požgan, F., Vanzo, A. Determination of thiol precursors in Sauvignon Blanc grapes. In: BIASIOLI, Franco (ed.). *Book of abstracts*. San Michele a/Adige - Italy: Research and Innovation Centre Fondazione Edmund Mach, 2013, str. 86-88.
http://eventi.fmach.it/MS-Food-Day/Program-and-abstracts

Grape extracts preparation and purification

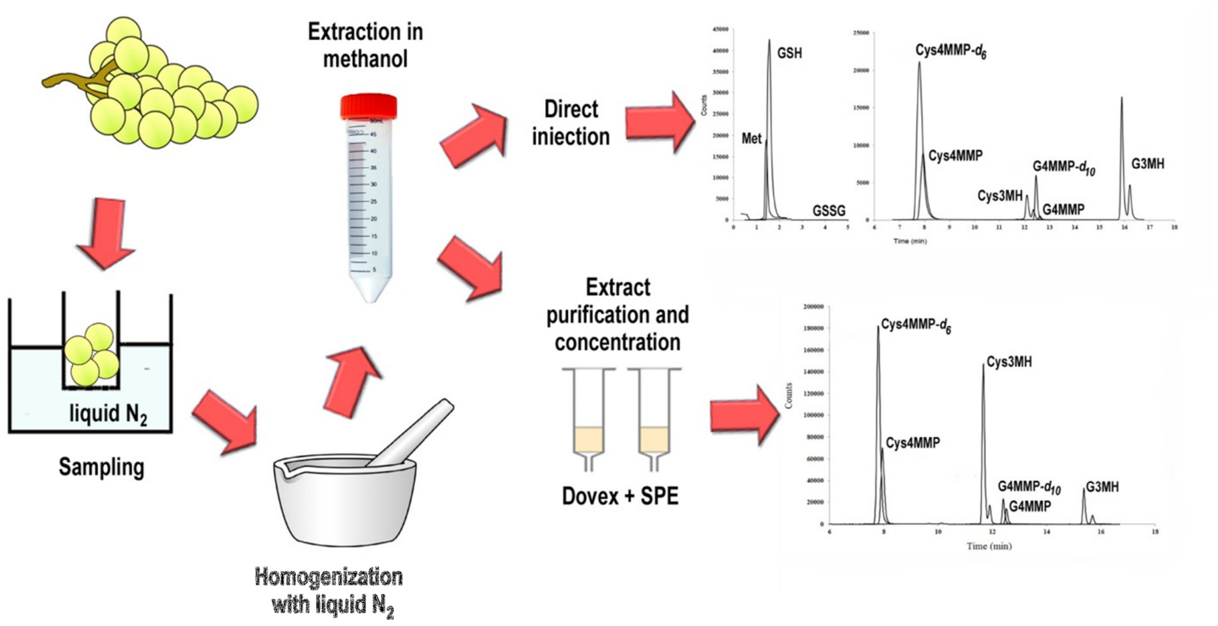

Figure S1: Schematic outline of sample preparation protocol.

Analysis of grapes

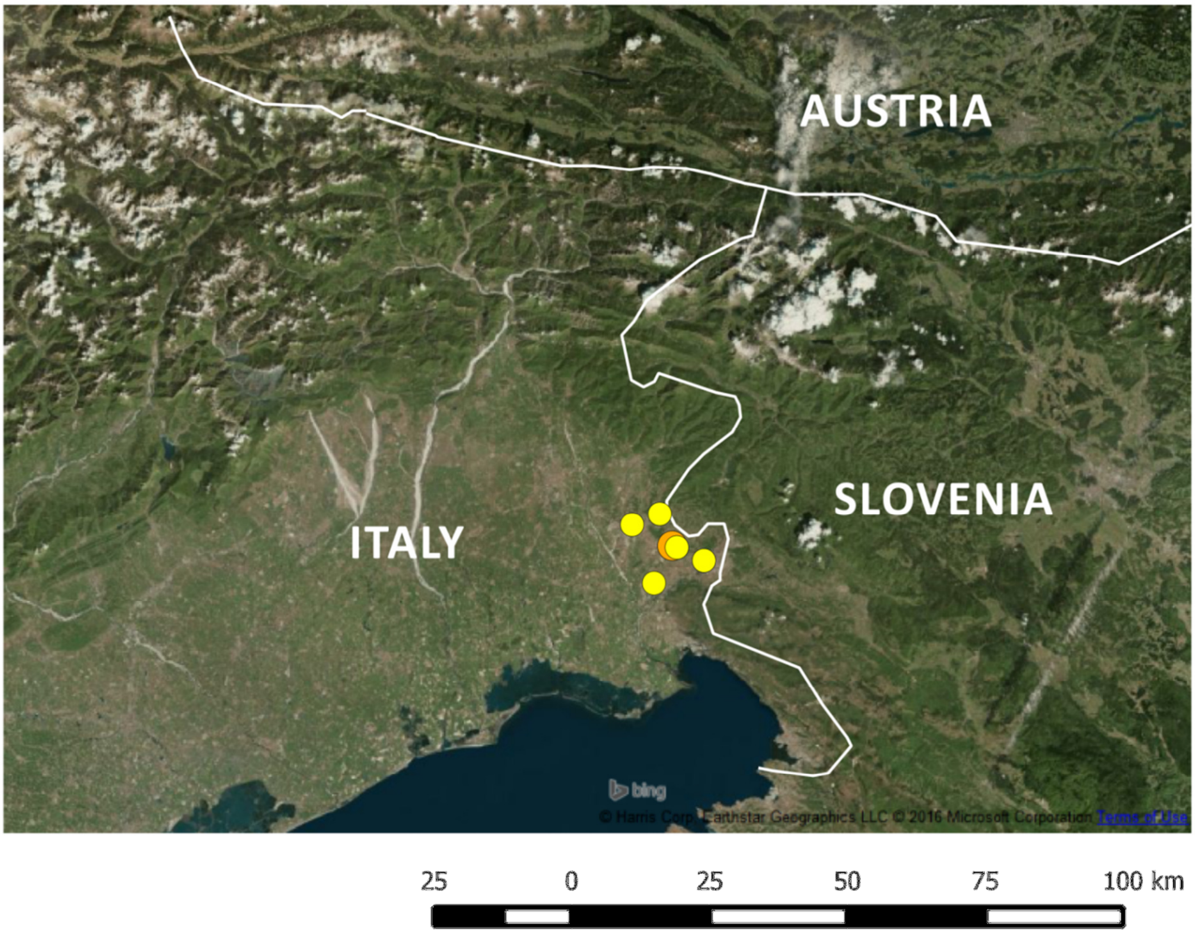

Figure S2. **Geografical position of the five sampled vineyards (yellow points) and weather station (orange point) in the North-Eastern part of Italy.** qGIS data processing (QGIS Development Team, 2013. QGIS Geographic Information System. Open Source Geospatial Foundation Project. http://www.qgis.org)

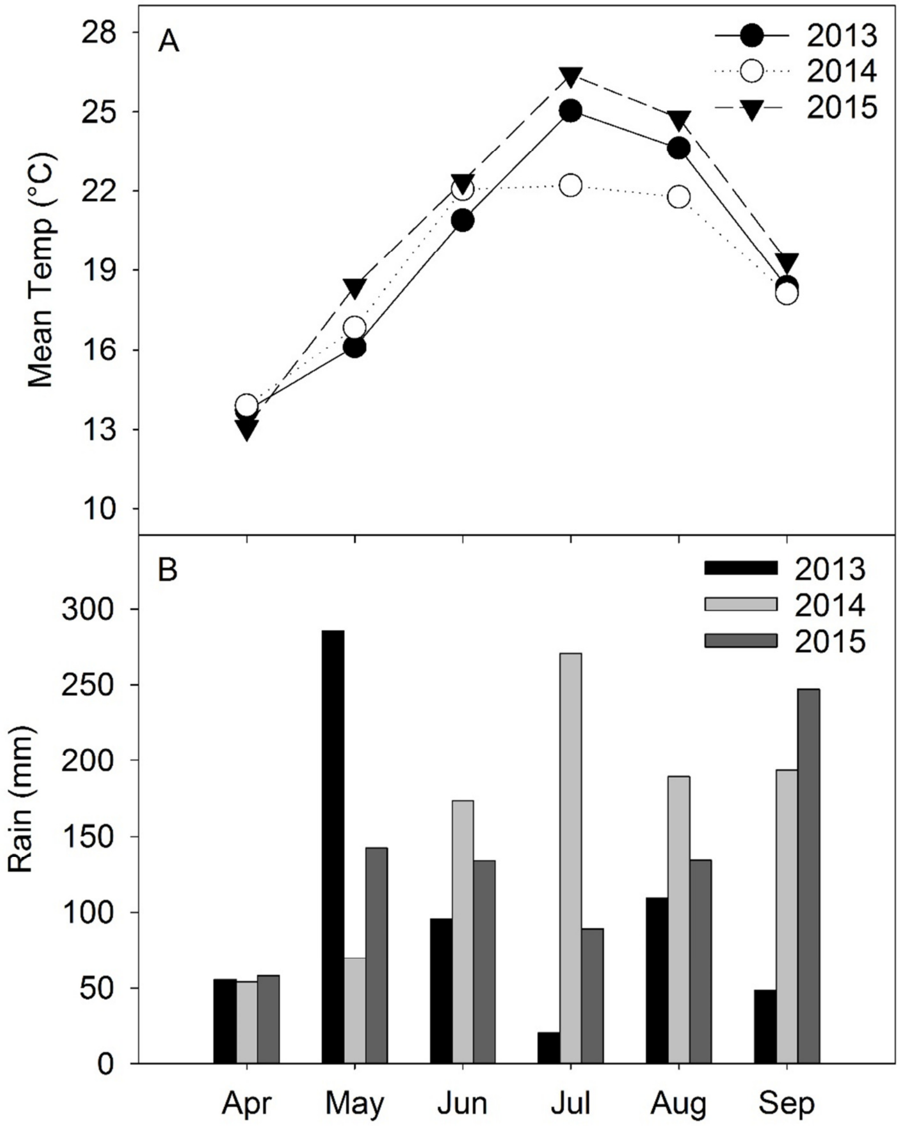

Figure S3. **Mean monthly temperatures (A) and cumulative monthly rain (B)** registered in the seasons 2013-15 in the weather stations of Capriva del Friuli in Italy (ARPA FVG - OSMER, http://www.meteo.fvg.it).

The difference in the temperatures during the growing season is important for the maturation of the grapes; Jones et al. (2012) reported that Sauvignon Blanc has a thermal requirement ranging between 1389 and 1667°C, in terms of Winkler index. In the season 2013 the grapes were harvested in a good condition of maturity (1552°C), while in 2014 the values of Winkler index approached the lower limit (1396°C). In the last season 2015, the index slightly overcame the upper limit of the grouping (1695°C). As related with the Winkler Index, we

understand that the trend of temperatures were also very different in the three years, and thus the distribution of the rain.

The trends of temperatures and distribution of rains were different in the three seasons considered in the study. In April the temperatures were similar in 2013 and 2014 and slightly lower in 2015, and the cumulative rain was nearly the same in all three years (Fig. S3). In May, the temperatures were much higher in 2015 (18.4°C) than in the other two seasons, and the same trend was observed in June. Thus, the beginning of the season was similar in the first two seasons in terms of temperatures, while quite different as regard rainfall; to date, the cumulate rain was the highest in May 2013 and in June 2014, while as opposite was the lowest in May 2014 and June 2013. In July and August, most of the differences appeared between the colder 2014 and both the other seasons in terms of temperatures, even if in the last year the temperatures were slightly higher as compared to 2013. As opposite, the rainfall was high in July (271 mm) and August 2014 (189 mm), and the largest differences among the seasons were observed in June, being the 2013 the year with the lowest rainfall (21 mm). The temperatures in September were still higher in the season 2015 and much more similar between the other two seasons. On the other hand, the cumulative rain was high in 2014 and mainly in 2015; during September 2014 the rain was well distributed during the month, while in the following 2015 two strong events of 96 and 62 mm accounted for the 64% of the total rain of September.

Jones, G. V; Reid, R.; Vilks, A. Climate, grapes, and wine: Structure and suitability in a variable and changing climate. In *The Geography of Wine: Regions, Terroir and Techniques*; Springer Netherlands, 2012; Vol. 9789400704, pp 109–133.

Methods

Synthesis of thiol precursors

3-*S*-glutathionylhexan-1-ol (G3MH)

^{1}H NMR (500 MHz, D_2O): δ = 0.86 (m, 3H), 1.38 (m, 2H), 1.54 (m, 2H), 1.72 (m, 1H), 1.84 (m, 1H), 2.14 (m, 2H), 2.51 (m, 2H), 2.83 (m, 2H), 3.05 (dd, J_1 = 5 Hz, J_2 = 13.5 Hz, 1H), 3.66–3.75 (m, 2H), 3.78 (t, J = 6.5 Hz , 1H), 3.93 (s, 2H), 4.54 (m, 1H) ppm.

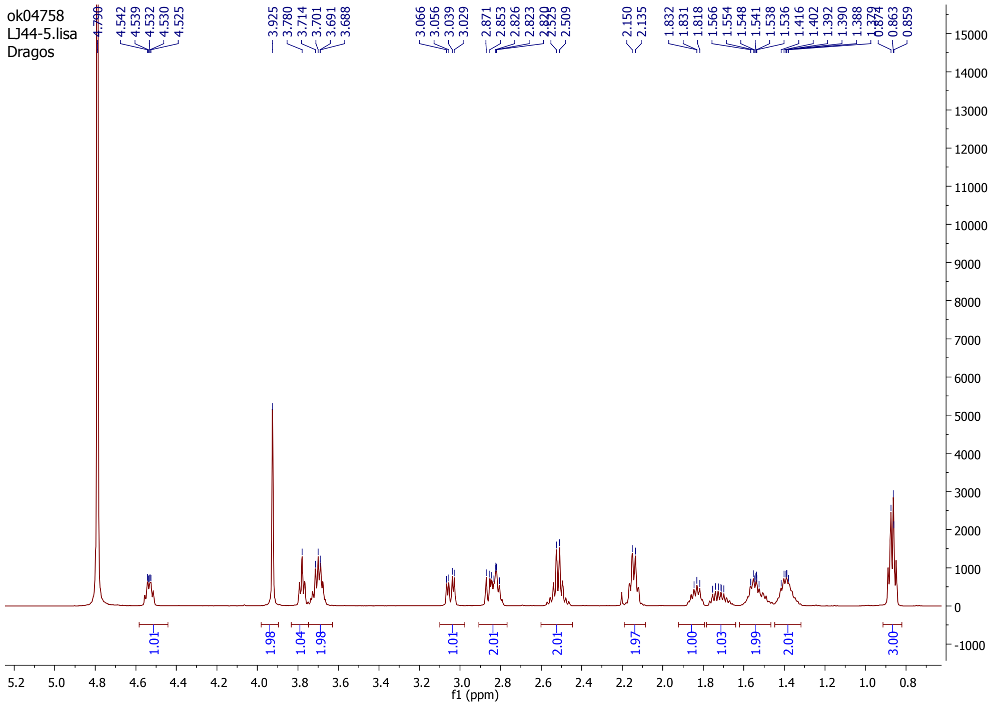

Figure S4: ^{1}H NMR spectra of G3MH.

HRMS (LC-TOF): $[M+H]^+$ calculated for $C_{16}H_{29}N_3O_7S$ 408.1799 Da, found 408.1800 Da.

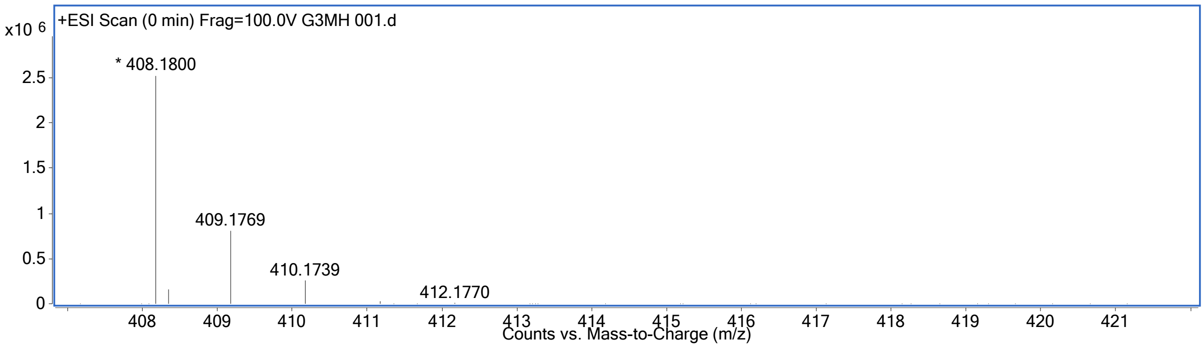

Figure S5: HR mass spectra (LC-TOF) of G3MH.

3-*S*-cysteinylhexan-1-ol (Cys3MH)

^{1}H NMR (500 MHz, D_2O): δ = 0.88 (t, *J* = 7.5 Hz, 3H), 1.40 (m, 2H), 1.56 (m, 2H), 1.71 (m, 1H), 1.86 (m, 1H), 2.88 (m, 1H), 3.00–3.18 (m, 2H), 3.71 (m, 2H), 4.06 (m, 1H) ppm.

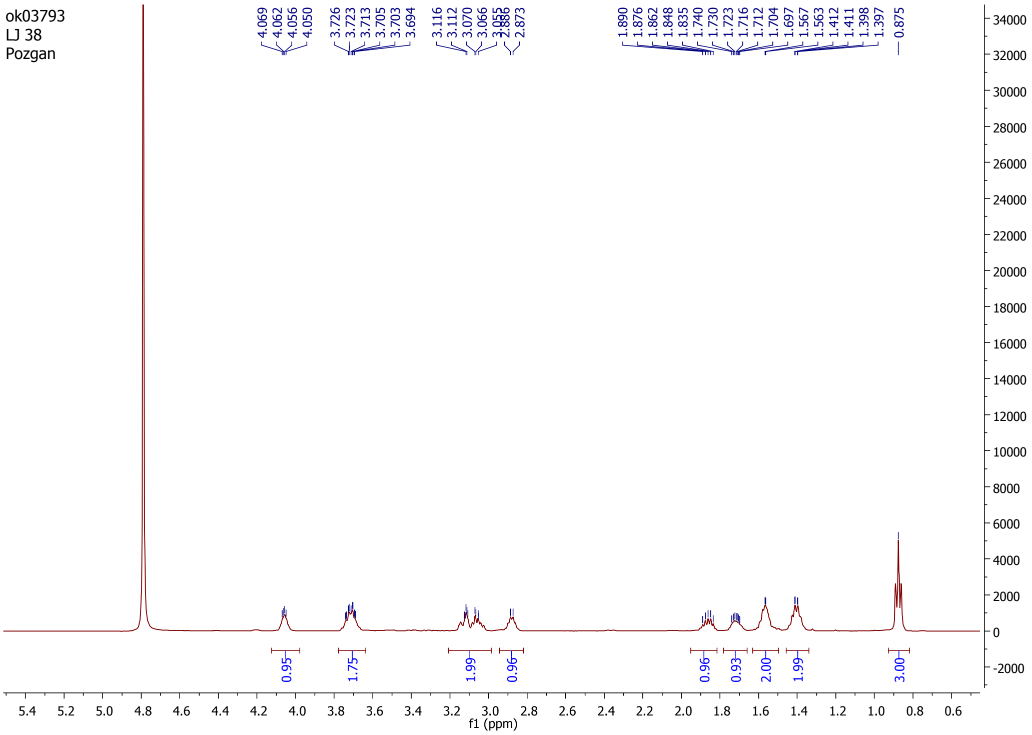

Figure S6: ^{1}H NMR spectra of Cys3MH.

^{19}F NMR (470 MHz, D_2O): δ = −75.6 ppm.

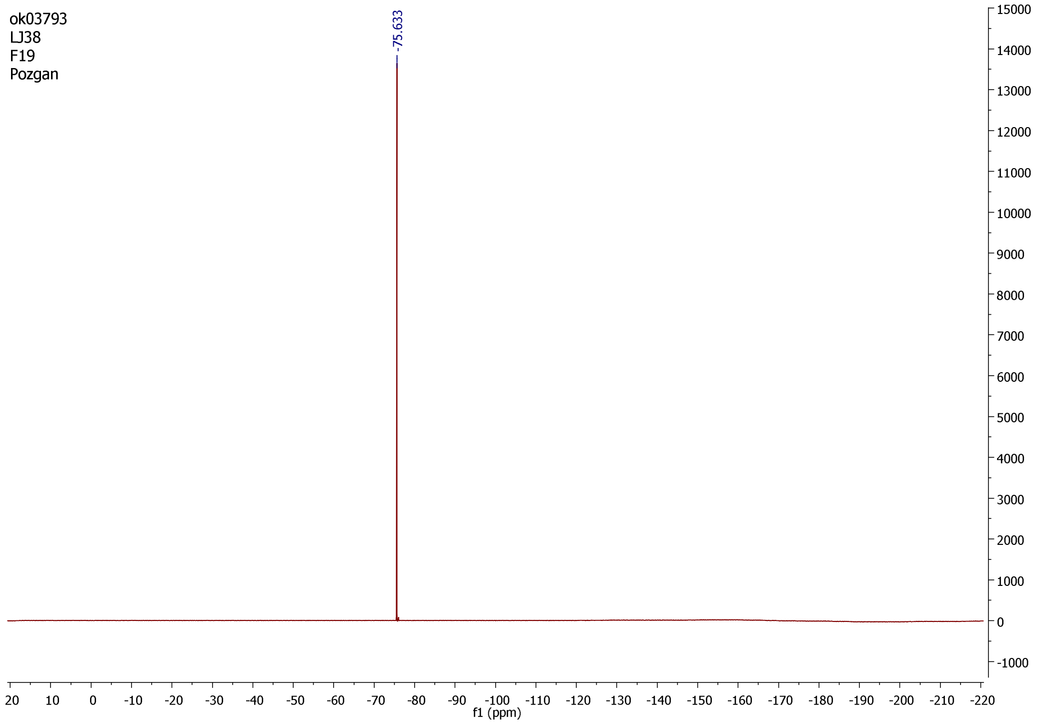

Figure S7: ^{19}F NMR spectra of Cys3MH.

HRMS (LC-TOF): $[M+H]^+$ calculated for $C_9H_{19}NO_3S$ 222.1158 Da, found 222.1149 Da.

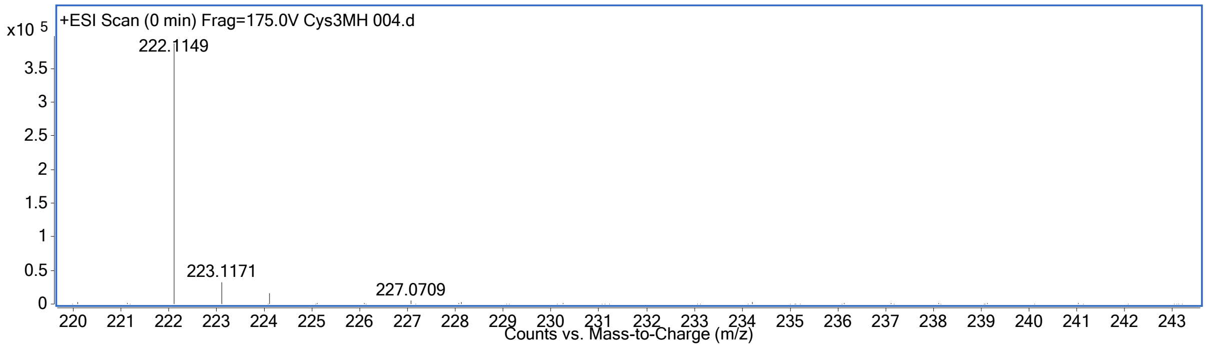

Figure S8: HR mass spectra (LC-TOF) of Cys3MH.

4-*S*-glutathionyl-4-methylpentan-2-one (G4MMP)

^{1}H NMR (300 MHz, D_2O): δ = 1.46 (s, 6H), 2.22 (m, 2H), 2.30 (s, 3H), 2.59 (m, 2H), 2.90 (s, 2H), 3.00 (dd, J_1 = 8.4 Hz, J_2 = 13.0 Hz, 1H), 3.15 (m, J_1 = 5.4 Hz, J_2 = 13.0 Hz, 1H), 3.88 (t, J = 6.3 Hz, 1H), 4.03 (s, 2H), 4.64 (dd, J_1 = 5.4 Hz, J_2 = 8.4 Hz , 1H) ppm.

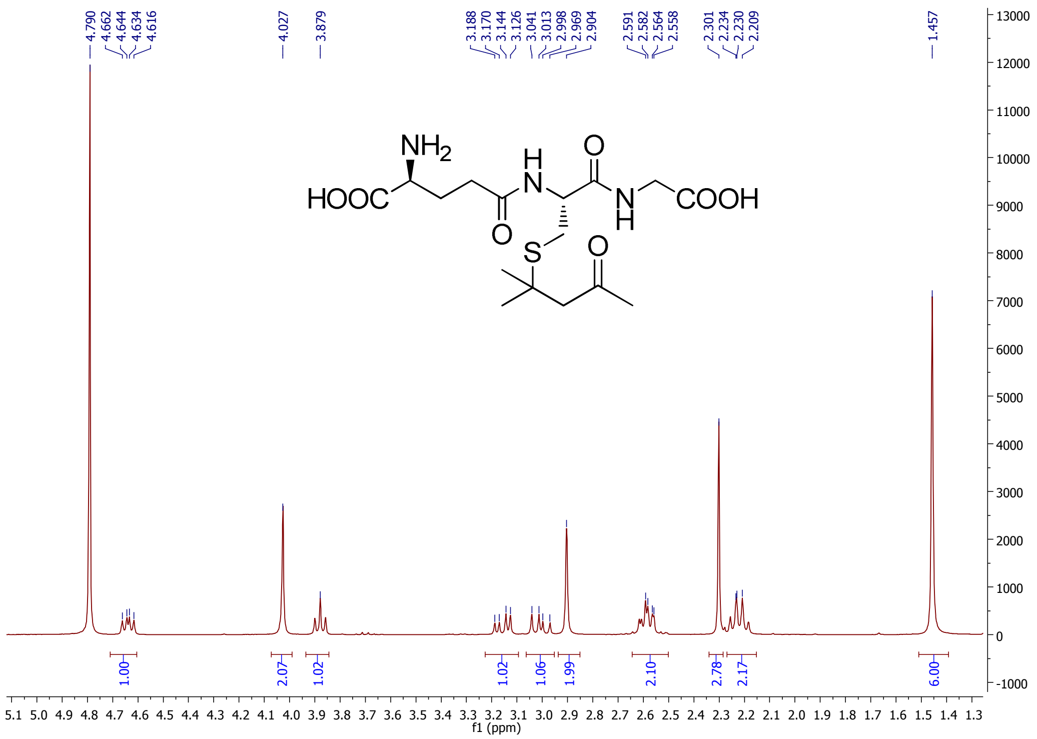

Figure S9: ^{1}H NMR spectra of G4MMP.

HRMS (LC-TOF): $[M+H]^+$ calculated for $C_{16}H_{27}N_3O_7S$ 406.1642 Da, found 406.1637 Da.

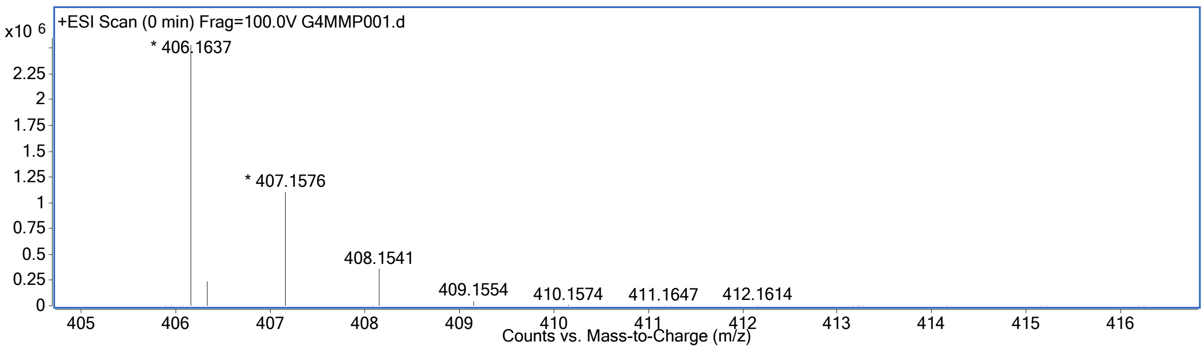

Figure S10: HR mass spectra (LC-TOF) of G4MMP.

[$^{2}H_{10}$] 4-*S*-glutathionyl-4-methylpentan-2-one (G4MMP-*d*$_{10}$).

^{1}H NMR (500 MHz, D_2O): δ = 2.14 (m, 2H), 2.51 (m, 2H), 2.81 (m, 1H), 2.93 (dd, J_1 = 8.5 Hz, J_2 = 13.0 Hz, 1H), 3.08 (dd, J_1 = 5.0 Hz, J_2 = 13.0 Hz, 1H), 3.79 (t, J = 6.3 Hz, 1H), 3.93 (s, 2H), 4.56 (dd, J_1 = 5.0 Hz, J_2 = 8.5 Hz , 1H) ppm.

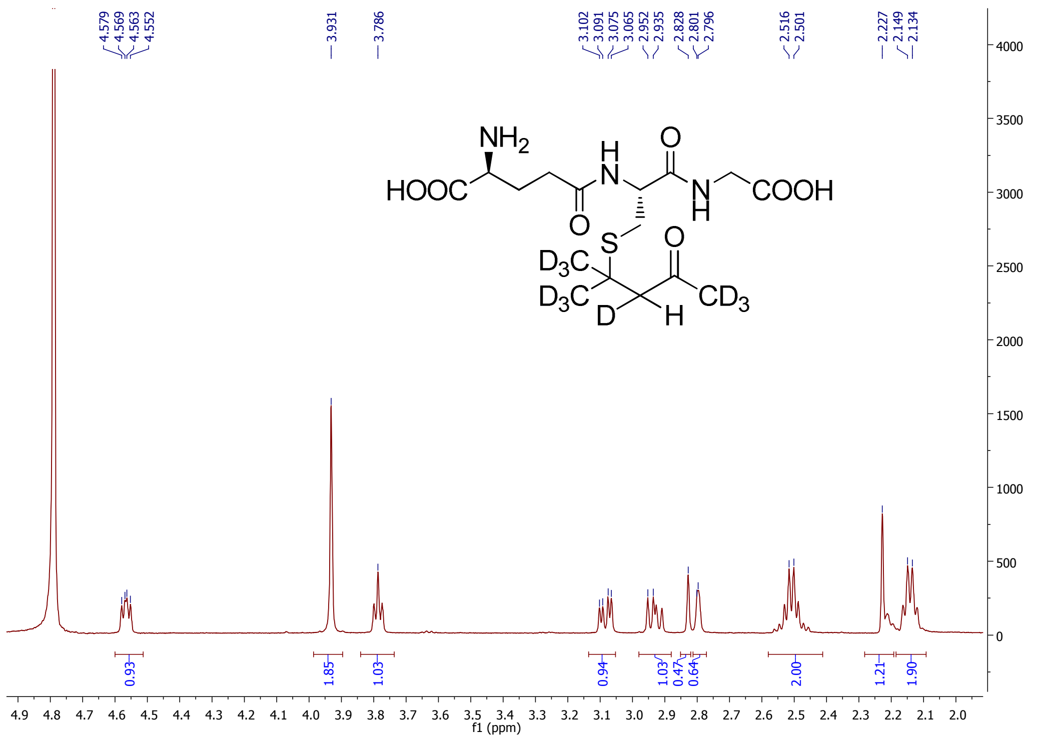

Figure S11: ^{1}H NMR spectra of G4MMP-*d*$_{10}$.

HRMS (LC-TOF): $[M+H]^+$ calculated for $C_{16}H_{17}N_3O_7SD_{10}$ 416.227 Da, found 416.2288 Da.

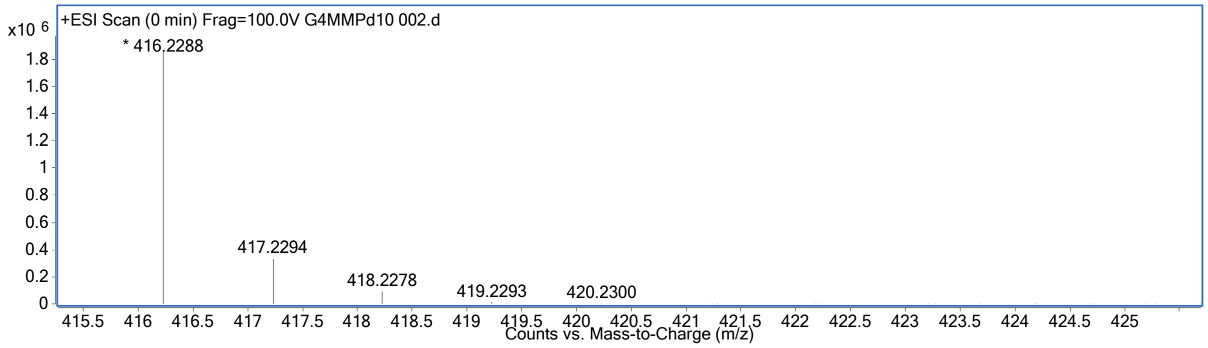

Figure S12: HR mass spectra (LC-TOF) of G4MMP-*d*$_{10}$.

4-*S*-cysteinyl-4-methylpentan-2-one (Cys4MMP).

^{1}H NMR (500 MHz, D_2O): δ = 1.41 (s, 6H), 2.24 (s, 3H), 2.86 (m, 2H), 3.09 (dd, J_1 = 7.5 Hz, J_2 = 14.0 Hz, 1H), 3.21 (dd, J_1 = 4.2 Hz, J_2 = 14.0 Hz, 1H), 4.11 (m, 1H) ppm.

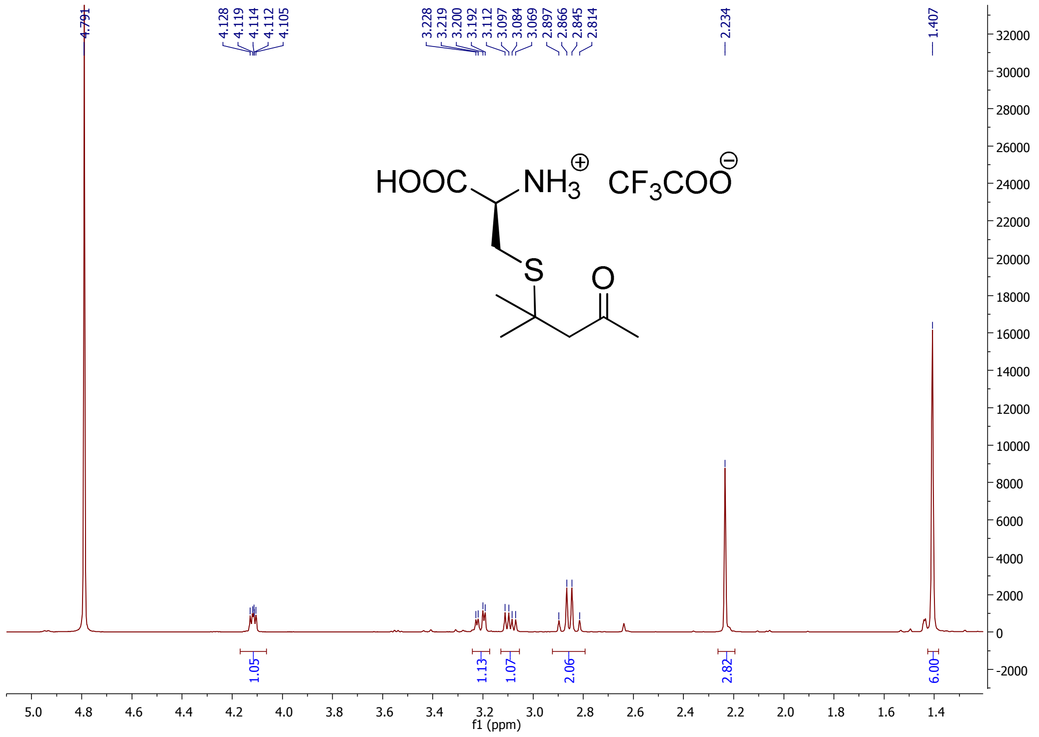

Figure S13: ^{1}H NMR spectra of Cys4MMP.

^{19}F NMR (470 MHz, D_2O): δ = −75.6 ppm.

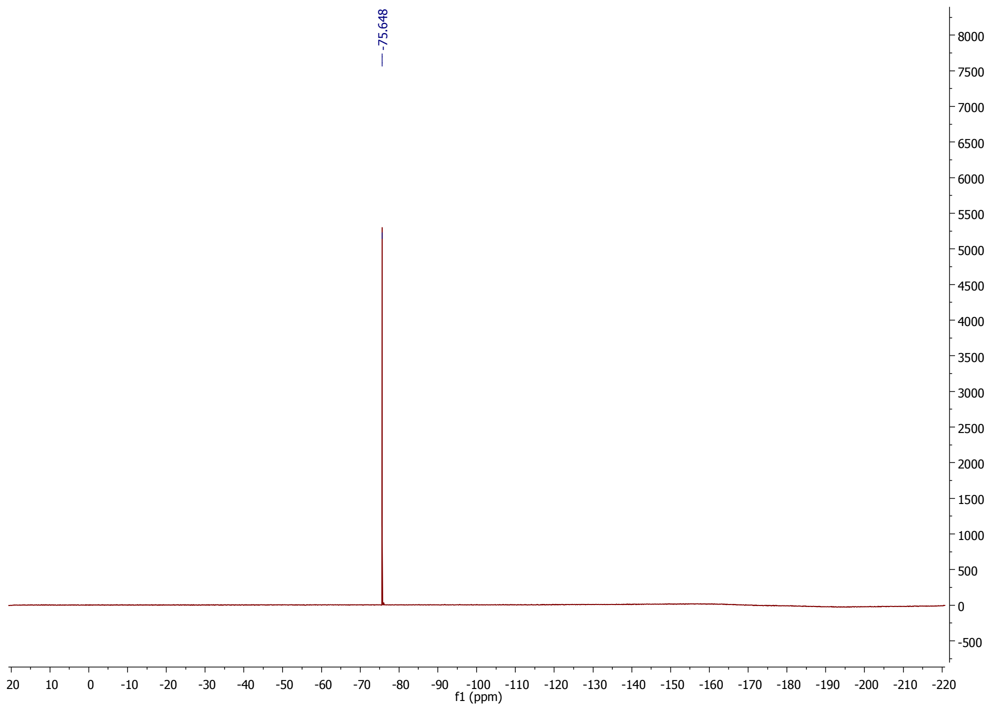

Figure S14: ^{19}F NMR spectra of Cys4MMP.

HRMS (LC-TOF): $[M+H]^+$ calculated for $C_9H_{17}NO_3S$ 220.1002 Da, found 220.1015 Da.

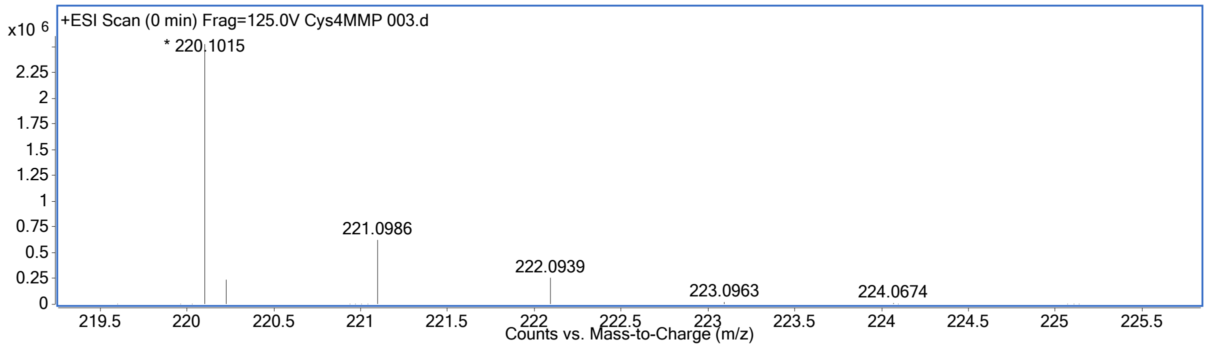

Figure S15: HR mass spectra (LC-TOF) of Cys4MMP.

[$^2H_6$] 4-*S*-cysteinyl-4-methylpentan-2-one (Cys4MMP-d_6).

^1H NMR (500 MHz, D_2O): δ = 2.22 (s, 3H), 2.78–2.90 (m, 2H), 3.05 (dd, J_1 = 7.5 Hz, J_2 = 13.5 Hz, 1H), 3.18 (dd, J_1 = 4 Hz, J_2 = 13.5 Hz, 1H), 3.96 (m, 1H) ppm.

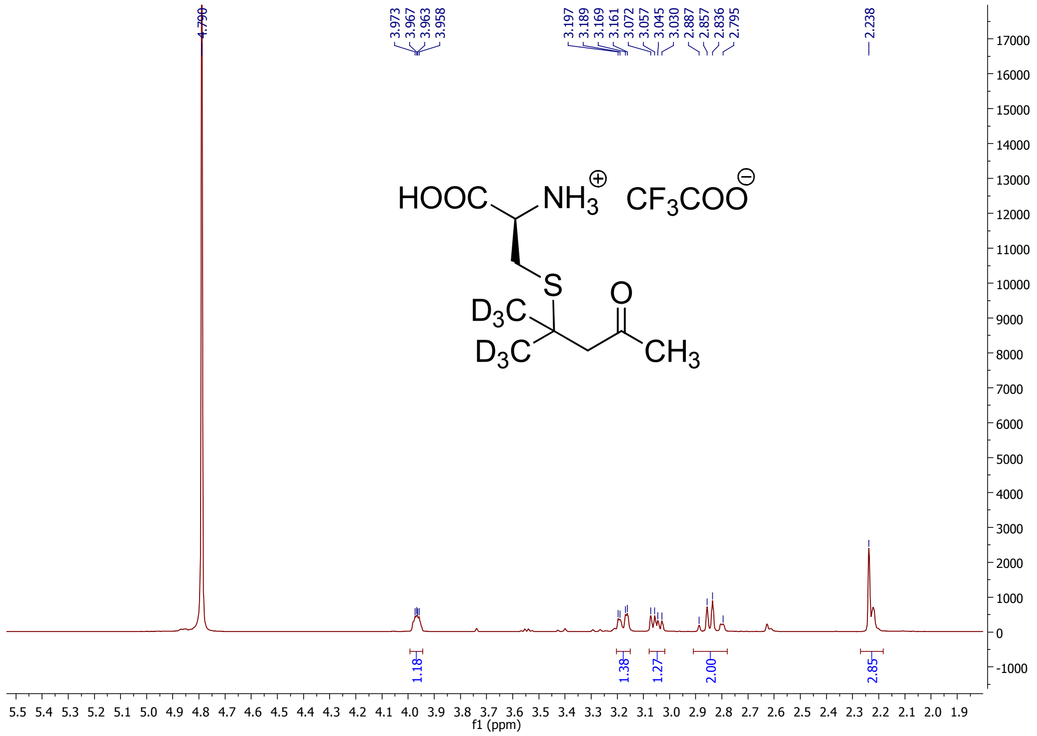

Figure S16: ^1H NMR spectra of Cys4MMP-d_6.

^{19}F NMR (470 MHz, D_2O): $\delta = -75.7$ ppm.

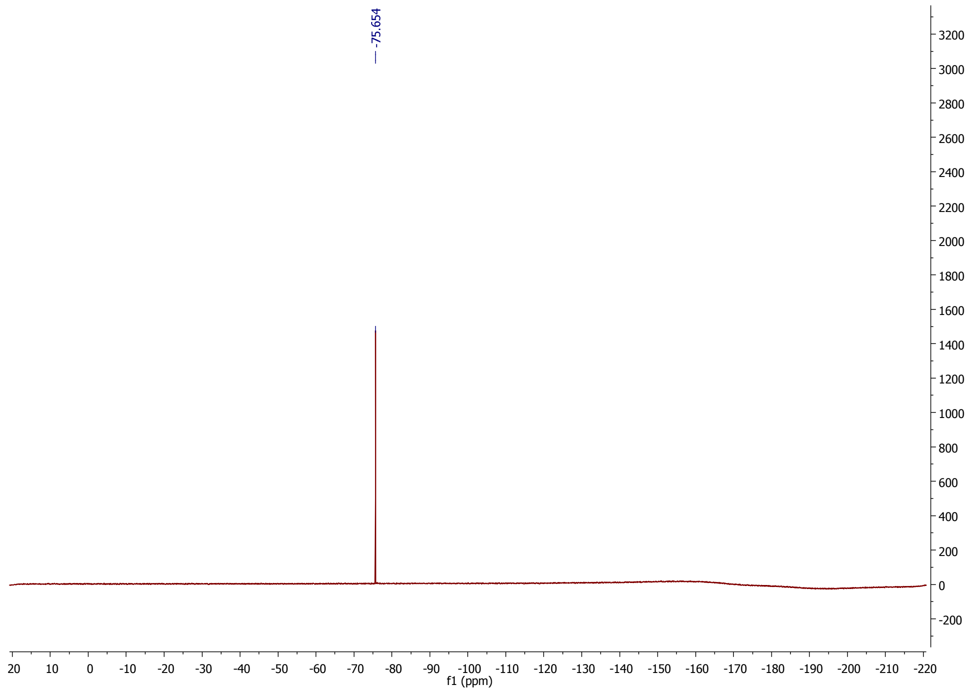

Figure S17: ^{19}F NMR spectra of Cys4MMP-d_6.

HRMS (LC-TOF): $[M+H]^+$ calculated for $C_9H_{11}NO_3SD_6$ 226.1379 Da, found 226.1402 Da.

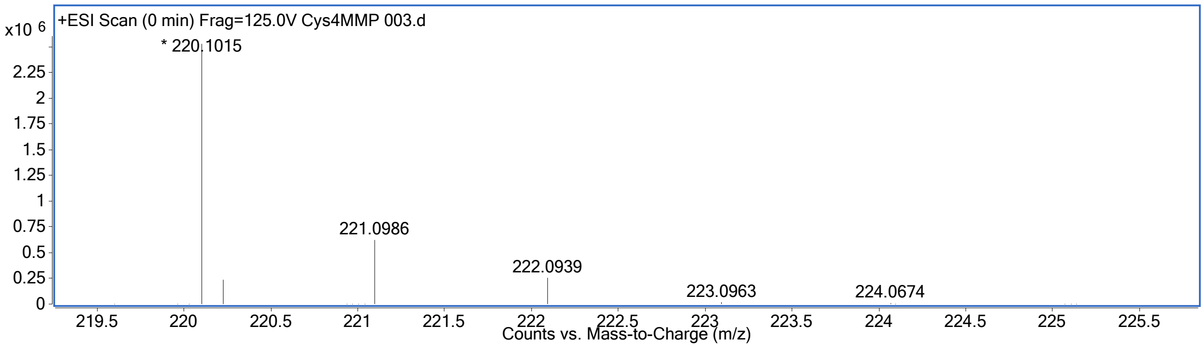

Figure S18: HR mass spectra (LC-TOF) of Cys4MMP-d_6.

Optimisation of extraction

	Met	GSH	GSSG	Cys4MMP	Cys3MH	G4MMP	G3MH
w/v	100% methanol						
1:9	540495	44531	23102	480	92415	529	508174
CV[a] (%)	1	25	10	15	7	104	8
1:4	474080	45141	39778	678	104843	273	594276
CV (%)	1	7	7	29	7	66	6
w/v	90% methanol in water						
1:9	529128	66218	55120	798	97067	211	571218
CV (%)	2	3	7	38	4	37	3
1:4	480086	60054	77486	1357	96566	146	570888
CV (%)	2	5	4	26	3	37	1

[a] Coefficient of variation

Table S2. The efficiency of extraction of L-methionine (Met), glutathione (GSH), oxidized glutathione (GSSG) and thiol precursors in 100% methanol and in 90% methanol in water at grape:solvent (w/v) ratios 1:4 and 1:9, respectively. The values represent the average peak areas (n=4) of compounds adjusted for dilution factor.
